# Sensory-Discriminative Three-Dimensional Body Pain Mobile App Measures Versus Traditional Pain Measurement With a Visual Analog Scale: Validation Study

**DOI:** 10.2196/17754

**Published:** 2020-08-19

**Authors:** Niko Kaciroti, Marcos Fabio DosSantos, Brenda Moura, Emily Light Bellile, Thiago Dias Nascimento, Eric Maslowski, Theodora E Danciu, Adam Donnell, Alexandre F DaSilva

**Affiliations:** 1 Center for Computational Medicine and Bioinformatics University of Michigan Ann Arbor, MI United States; 2 Headache & Orofacial Pain Effort (H.O.P.E.) Department of Biologic and Materials Sciences School of Dentistry, University of Michigan Ann Arbor, MI United States; 3 Department of Biostatistics School of Public Health University of Michigan Ann Arbor, MI United States; 4 Instituto de Ciências Biomédicas (ICB) Universidade Federal do Rio de Janeiro (UFRJ) Rio de Janeiro Brazil; 5 Programa de Pós-Graduação em Medicina (Radiologia) Universidade Federal do Rio de Janeiro (UFRJ). Rio de Janeiro Brazil; 6 Department of Public Health University of Michigan Ann Arbor, MI United States; 7 Moxytech Inc Ann Arbor, MI United States; 8 Department of Periodontics and Medicine School of Dentistry University of Michigan Ann Arbor, Michigan, MI United States; 9 Orthodontics Department of Developmental Biology Harvard University Boston, MA United States

**Keywords:** pain measurement, chronic pain, migraine, visual analog scale, facial pain

## Abstract

**Background:**

To quantify pain severity in patients and the efficacy treatments, researchers and clinicians apply tools such as the traditional visual analog scale (VAS) that leads to inaccurate interpretation of the main sensory pain.

**Objective:**

This study aimed to validate the pain measurements of a neuroscience-based 3D body pain mobile app called GeoPain.

**Methods:**

Patients with temporomandibular disorder (TMD) were assessed using GeoPain measures in comparison to VAS and positive and negative affect schedule (PANAS), pain and mood scales, respectively. Principal component analysis (PCA), scatter score analysis, Pearson methods, and effect size were used to determine the correlation between GeoPain and VAS measures.

**Results:**

The PCA resulted in two main orthogonal components: first principal component (PC1) and second principal component (PC2). PC1 comprises a combination score of all GeoPain measures, which had a high internal consistency and clustered together in TMD pain. PC2 included VAS and PANAS. All loading coefficients for GeoPain measures in PC1 were above 0.70, with low loadings for VAS and PANAS. Meanwhile, PC2 was dominated by a VAS and PANAS coefficient >0.4. Repeated measure analysis revealed a strong correlation between the VAS and mood scores from PANAS over time, which might be related to the subjectivity of the VAS measure, whereas sensory-discriminative GeoPain measures, not VAS, demonstrated an association between chronicity and TMD pain in locations spread away from the most commonly reported area or pain epicenter (*P*=.01). Analysis using VAS did not detect an association at baseline between TMD and chronic pain. The long-term reliability (lag >1 day) was consistently high for the pain area and intensity number summation (PAINS) with lag autocorrelations averaging between 0.7 and 0.8, and greater than the autocorrelations for VAS averaging between 0.3 and 0.6. The combination of higher reliability for PAINS and its objectivity, displayed by the lack of association with PANAS as compared with VAS, indicated that PAINS has better sensitivity and reliability for measuring treatment effect over time for sensory-discriminative pain. The effect sizes for PAINS were larger than those for VAS, consequently requiring smaller sample sizes to assess the analgesic efficacy of treatment if PAINS was used versus VAS. The PAINS effect size was 0.51 SD for both facial sides and 0.60 SD for the right side versus 0.35 SD for VAS. Therefore, the sample size required to detect such effect sizes with 80% power would be n=125 per group for VAS, but as low as n=44 per group for PAINS, which is almost a third of the sample size needed by VAS.

**Conclusions:**

GeoPain demonstrates precision and reliability as a 3D mobile interface for measuring and analyzing sensory-discriminative aspects of subregional pain in terms of its severity and response to treatment, without being influenced by mood variations from patients.

## Introduction

### Background

The true complexity of a given pain area and intensity and pain progression are never precisely documented at individual, longitudinal, and epidemiologic levels. Hence, crucial information in pain investigation and treatment is inexorably lost. This is especially true when patients have chronic and overlapping pain conditions, where pain is refractory to treatment and usually transverse or co-exist in multiple locations and dermatomes with different intensities. Research studies from the past 10 years have identified a large overlap between a number of chronic pain conditions, including temporomandibular disorder (TMD), migraine, neuropathic pain, fibromyalgia, irritable bowel syndrome, and so forth [1]. In fact, the presence of multiple large body areas with pain is associated with worse pain experience and prognosis [2-5]. Nonetheless, patients are frequently asked to represent their overall pain by giving a single number (numerical rating scale, NRS), verbally state its level (verbal rating scale, VRS), or similarly mark on a 10 cm line their pain level (visual analog scale, VAS). Despite being considered the gold standard in clinical trials for pain, traditional measurement tools have huge disadvantages, including low precision and higher rates of incorrect responses [6,7]. Perhaps, the most important aspect is the assumption that overall pain is a unidimensional experience that can be measured with a single-item scale [8,9]. As per ClinicalTrials.gov data, research trials for pain killers have increased at an average rate of greater than 20% in the past 15 years. Despite the rise in costs and boost in investments, medical assessments of patients' pain and the response to current and potential novel analgesic therapies are mostly based on subjective tools, such as grading of pain on a numerical scale. More objective approaches are needed to precisely measure and track in real time, more single and overlapping pain conditions, their evolution, and the therapeutic approaches best suited for treating pain in an individual and in large populations. The bottom line is that sensory pain assessment allows clinicians and scientists to monitor the longitudinal severity of the pain disorder and to quantify analgesic treatment effects [10], independent of the emotional and cognitive impact. One missing disconnect of the clinical assessment of pain from the years of pain neuroimaging research is that the sensory-discriminative aspect of pain is not only processed by the brain for its intensity but also for its accurate account of region and even subregional pain location and area. Together with other pain-related brain structures, the primary somatosensory cortex (S1) ultimately processes pain based on the homuncular noxious ascending inputs from multiple anatomic subregions [11,12].

To address the inaccuracy issues related to the main sensory pain scales traditionally used in the clinical and research practice, a collaborative effort from pain neuroimaging and 3D experts at the University of Michigan has developed a 3D body mobile in-house and optimized it for multiple pain disorders to better match the objectivity of pain neuroimaging and neuromodulation studies [13]. GeoPain (licensed by MoxyTech Inc) is a free or customized mobile app for tracking, analyzing, and communicating pain on multiple mobile platforms. Please, refer to Multimedia Appendix 1 (flowchart of the patients included in the clinical trial [13]) and to Multimedia Appendix 2 (demographic data of patients included in the clinical trial [13]). It is a reproducible and quantifiable 3D navigation system of grids that generates clinical and research tools for single and overlapping pain disorders by providing detailed sensory-discriminative measurements for the full body and by subregions. The personalized interface allows the patient to quickly delineate the intensity and area of pain on diverse rotating 3D body models (different gender and age avatars) by simply touching and zooming on the screen to where it hurts using a touch device such as touch-screen desktops, mobile phones, or tablets. The AI-enabled time-stamped technology precisely and quantitatively records and communicates their pain(s) and associated symptoms, which better mirrors the way the brain decodes pain severity across the body. For instance, neuroimaging studies have reported that the level of endogenous μ-opioid activation in episodic and chronic migraine patients is highly affected by the pain area and intensity number summation (PAINS) [14], one of the main sensory pain measurements provided by GeoPain [14-16]. However, no correlations were found with μ-opioid receptor binding based on the attack pain intensity or area separately, or the traditional VAS score. In our high-definition neuromodulation study targeting the unilateral primary motor cortex, a significant pain difference between sham and active groups was detected 1 month before the traditional VAS by accurately looking at subregional pain area, intensity, and both sensory-discriminative measures combined (PAINS) [13].

### Objectives

Following these multiple neuroimaging and neuromodulation studies, we aimed to specifically validate sensory-discriminative GeoPain measurements from our TMD neuromodulation trial [13] and better understand their reliability to assess sensory pain impact and sample size of patients needed compared with the traditional VAS score.

## Methods

### Study Design

The study was a randomized, placebo-controlled, blinded clinical trial of high-definition transcranial direct current stimulation (HD-tDCS) of the motor cortex in 24 female patients with chronic TMD. The data obtained has been used to assess the validity, reliability, and utility of GeoPain measures in comparison to the VAS. The results of the clinical trial, which are not the aim of this study, have already been published (Trial Registration: Clinicaltrials.gov NCT02247063) [13].

After being assigned to the active or sham HD-tDCS group, participants presented during week 1 for a baseline visit. The protocol included 5 days of stimulation, a 1-week follow-up, and 1-month follow-up. The pain measures VAS (rated from 0-10), short form of the McGill Pain Questionnaire, and GeoPain app (initially released as PainTrek) were performed, thereby allowing tracking of the effectiveness of the treatment. Subjects’ measures derived from GeoPain included PAINS were collected. The positive and negative affect schedule (PANAS) was used to assess mood changes. The University of Michigan Institutional Review Board approved the study (HUM00070766) and written informed consent was obtained from all participants.

### GeoPain Technology

**Figure 1:**
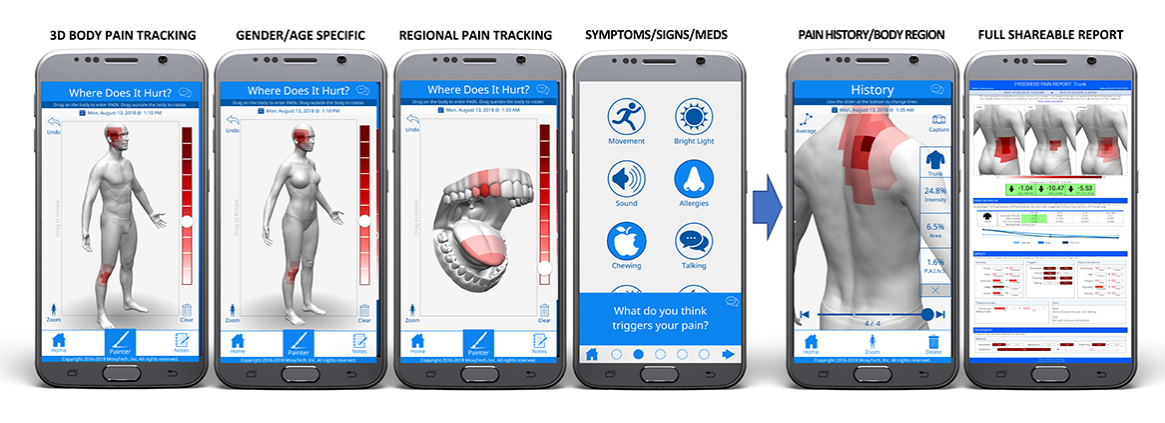
GeoPain tracks and measures pain in diverse single or group 3D-body models (gender and age group) and across multiple body locations. Whether the patient has TMD, migraine, fibromyalgia, or dental pain, the app can measure if a particular medication or clinical procedure is effective for each localized or spread pain condition. TMD: temporomandibular disorder.

GeoPain is a free stand-alone and embedded mobile app developed in collaboration with the Headache and Orofacial Pain Effort (HOPE) at the University of Michigan and is currently licensed by the spinoff MoxyTech Inc. This pain app is available for free on Google Play and the Apple App Store. GeoPain provides a 3D body map based on a squared grid system with vertical and horizontal coordinates using anatomical landmarks. Each quadrangle, measuring approximately 1.6 cm × 1.6 cm, frames well-detailed 3D body regions, such as trunk, extremities (arms and hands, legs and feet), and craniofacial and intra oral areas for the patient to express his or her exact global and sectional pain location and intensity, as well as signs and symptoms (Figure 1).

At each session, using the app on an iPad (Apple Inc), participants drew their pain in multiple shades of red on the touch-sensitive screen ranging from pink (mild pain), red (moderate pain), and dark red (severe pain). It is also categorized with *mild*, *moderate,* and *severe* to provide some guidance for their selection and follows the accepted grouping mentioned next. Average pain is the average score of all cells that are marked as painful, with a scale of 1-3 (mild=1, moderate=2, severe=3). Pain area was the percent of the area of the head and neck region that was experiencing pain, with a scale of 0%-100% of all cells. The general size of the cells is about right for the adult male model; however, those sizes will scale depending on the body type chosen but remain accurate relative to anatomical landmarks. Finally, PAINS was the cumulative score for the cells. On the GeoPain version for this particular trial, there were a total of 2026 cells across the body. The head and neck have 382, and just the head has 322. The cells of the body are broken into 54 regions; 27 if you do not distinguish between left and right sides of the body. The average pain is the normal average for a particular region or full body. GeoPain takes all cells in a specified range, adds their intensities together, and then divides that by the number of entries. For the three pain measures, the analysis was performed for the entire head and neck area, or unilaterally, to understand how sensory-discriminative pain measures changed ipsilateral or contralateral to the putative primary cortex (M1) stimulation.

### Statistical Methods

Principal component analysis (PCA) was used to determine the correlation structure among the variables: PAINS, average pains, maximum pain, area of pain, VAS, PANAS. To assess the test-retest reliability of the PAINS and VAS measures over time, the autocorrelation between neighboring time points (ranging from 1 to 40 days) was performed. These lag autocorrelations were estimated using Pearson methods. Smoothing splines were used to model the time trend of such autocorrelations for both PAINS and VAS. All statistical analyses were performed using SAS version 9.4 (SAS Institute Inc). Cohen’s effect size for both the PAINS and VAS were calculated for the delta change at 1 month from baseline to assess the longer term (1-month post-treatment) sensitivity of each measure for evaluating the treatment efficacy.

## Results

### Principal Component Analysis: GeoPain Versus Visual Analog Scale

**Figure 2:**
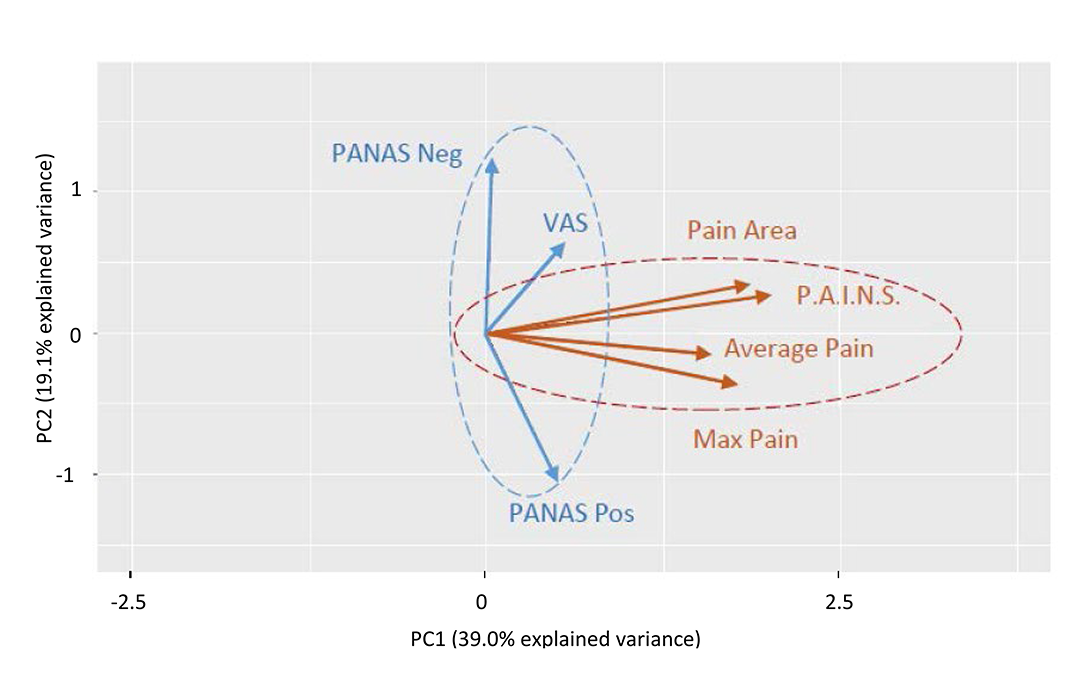
PCA graph showing the most representative correlation among the measures at the baseline visit. PAINS: pain area and intensity number summation; PANAS: positive and negative affect schedule; PCA: principal component analysis; PC1: first principal component; PC2: second principal component; VAS: visual analog scale.

**Figure 3:**
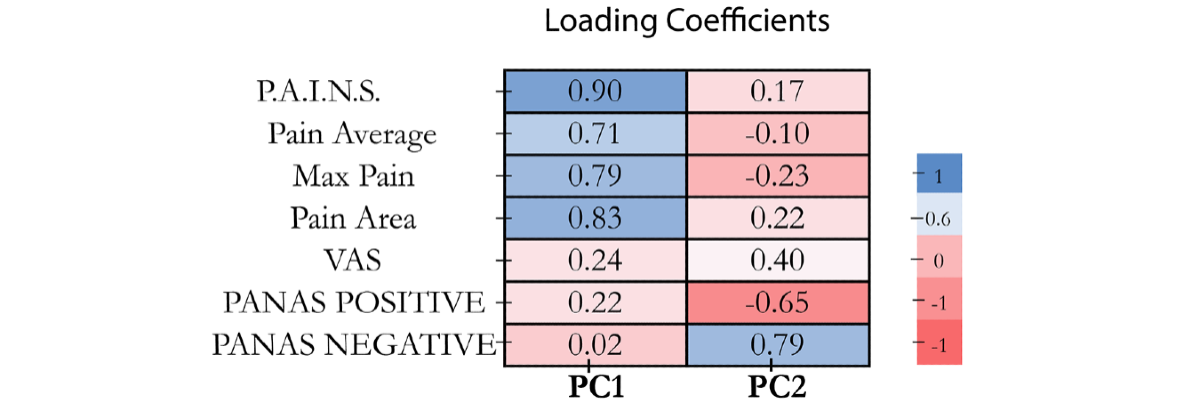
PC1 shows the correlation among GeoPain measures, and PC2 shows a more relevant correlation between VAS and PANAS on the baseline visit. The darker colors indicate higher loading coefficients. PAINS: pain area and intensity number summation; PANAS: positive and negative affect schedule; PC1: first principal component; PC2: second principal component; VAS: visual analog scale.

PCA resulted in two main orthogonal components. The first principal component (PC1) comprises a combination score of all PAINS measures which had a high internal consistency and clustered together in TMD pain (Figure 2). The second principal component (PC2) included VAS and PANAS. The loading coefficients for each variable in PC1 and PC2 are shown in Figure 3. All loading coefficients for PAINS measures in PC1 were above 0.70, with low loadings for VAS and PANAS. The loading coefficient for PC2 was dominated by VAS and PANAS all >0.4. The biplot shown in Figure 2 shows that PC1 dominated by the GeoPain measures added is approximately orthogonal to PANAS, whereas PC2 comprises VAS along with PANAS.

### Pain Epicenter: Correlating Duration of Pain with GeoPain and Visual Analog Scale Measures

**Figure 4:**
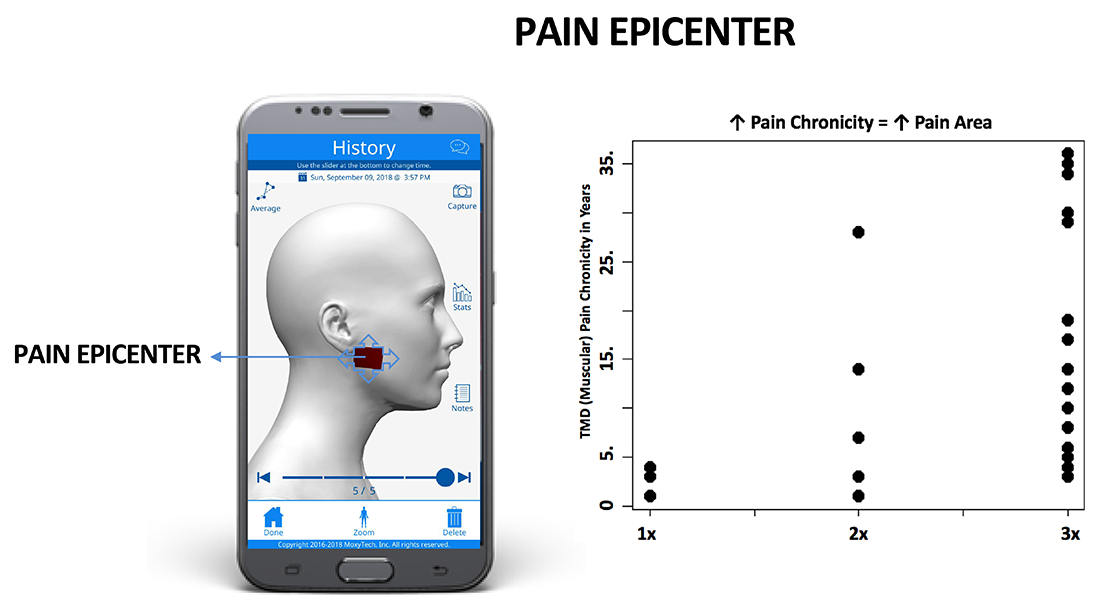
GeoPain measures demonstrated an association between chronicity and pain in locations farther away from the pain epicenter (*P*=.01). Analysis using VAS detected neither an association at baseline for pain area nor chronicity. TMD: temporomandibular disorder; VAS: visual analog scale.

GeoPain measures demonstrated an association between chronicity and pain in locations further away from the most commonly reported area, or pain epicenter, in the chronic TMD cohort (*P*=.01). We found a significant relationship between pain duration and further spread from pain epicenter cells using scatter score analysis (Figure 4). There was no correlation between VAS and pain duration in our patients.

### Clinical Trial: Delta Change and Effect Sizes Based on Pain Area and Intensity Number Summation and Visual Analog Scale

**Figure 5:**
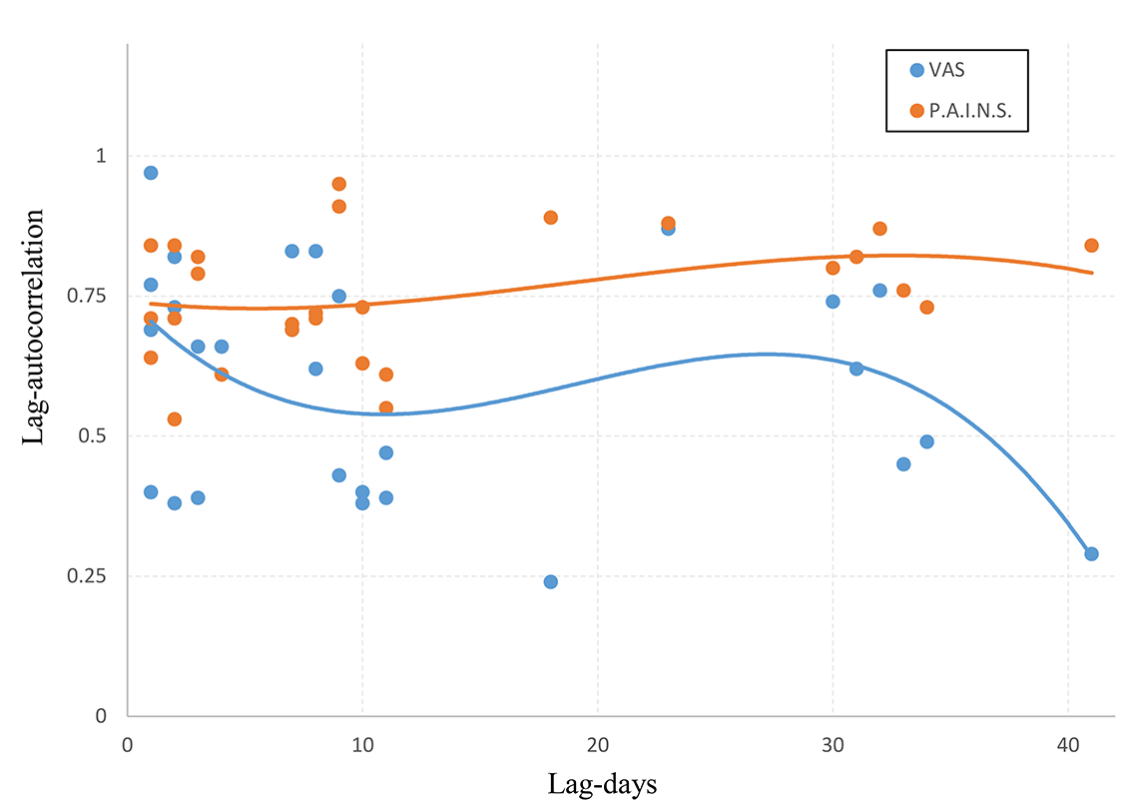
Correlation over time between PAINS and VAS scores. The short-term 1-day reliability for PAINS (red) and VAS (blue) scores were similar. However, longer term reliability is consistently high for PAINS and higher than that for VAS on average. PAINS: pain area and intensity number summation; VAS: visual analog scale.

GeoPain (PAINS) showed consistent test-retest reliability compared with VAS, demonstrated by higher lag-auto correlations across the entire clinical trial data. Figure 5 displays the within-measure autocorrelation between neighboring time points from 1 to 40 days for PAINS and VAS. The short-term 1-day reliability for PAINS and VAS was similar and high, with an autocorrelation of around 0.7. However, longer term reliability (>1 day) is consistently high for PAINS averaging between 0.7 and 0.8, and higher than the autocorrelations for VAS averaging between 0.3 and 0.6. The combination of higher reliability for PAINS and its objectivity displayed by the lack of association with PANAS as compared with VAS suggests that PAINS demonstrates a potential better sensitivity for measuring treatment effect over time for sensory-discriminative pain.

**Figure 6:**
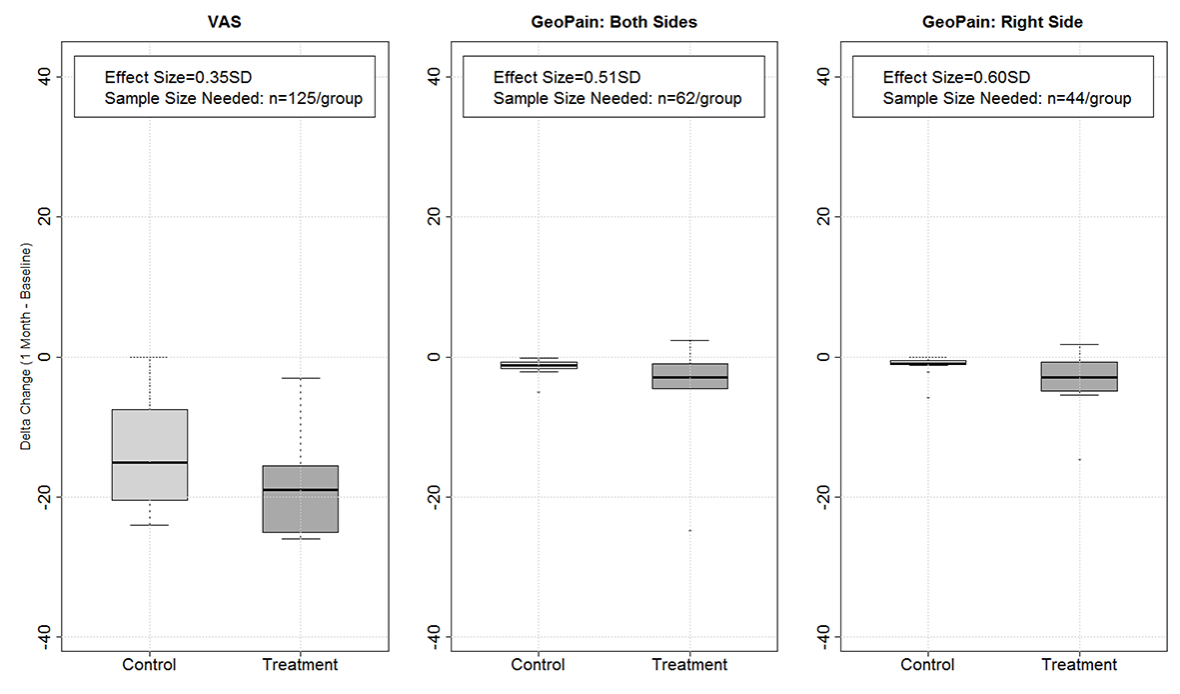
Graphs showing the power between the GeoPain × VAS. This figure shows the box plot for delta change by treatment and the corresponding effect sizes based on PAINS and VAS. The effect sizes for PAINS are larger than those for VAS, consequently requiring smaller sample sizes to assess the utility of treatment if PAINS is used versus VAS. Specifically, the effect sizes for PAINS were 0.51 SD for both regions and 0.60 SD for right side versus 0.31 SD for VAS. Thus, the sample size required to detect such effect sizes with 80% power would be n=145 per group for VAS, but for PAINS n=62 per group if both sides were used, n=44 per group for the right side, and n=82 per group for the left. PAINS: pain area and intensity number summation; VAS: visual analog scale.

The GeoPain measure is a more specific sensory-discriminative measure of pain compared with the VAS, resulting in higher statistical power. For example, we were able to detect significant differences by treatment during the 5-day trial period using GeoPain but not when VAS was used. Higher differences were also observed using GeoPain during the 1-month follow-up after the trial. On the basis of the study [13], to achieve a statistical power of 80% when using VAS versus GeoPain a sample size twice or thrice as high for 1 month comparisons from the traditional 0 to 10 pain measurement method is required (Figure 6).

## Discussion

### Principal Findings

This study specifically assessed and validated the effectiveness of 3D−body pain measurements from a free mobile appl, called GeoPain, compared with VAS in a clinical trial with chronic TMD patients following sham or active 5-daily M1 HD-tDCS neuromodulation sessions. The results showed that the sensory-discriminative GeoPain measures, which included global and subregional pain area, intensity (average and maximum pain), and their combination (PAINS), were consistent and clustered together in TMD pain at baseline. On the contrary, VAS scores were bidirectionally correlated with swinging in patients’ PANAS. In addition, the more chronic the pain in years, the larger the pain area spread from its epicenter. During the clinical trial (1 to 40 days), the long-term reliability was also steadily high for PAINS and low for VAS. Subsequently, the effect size for PAINS was larger than that for VAS, and as a result, half to a third of sample sizes of patients are needed to evaluate a particular pain-relief therapy if PAINS is used compared with VAS.

### Patients’ Positive Mood Influences Visual Analog Scale Pain Scores

Previous studies have demonstrated that unidimensional numerical scales such as the NRS or VAS provide a false impression of being sensitive and reliable measurements of pain performed in millimeters or numerals [17-19], and also lead to inaccuracies and biases. In addition, a large body of literature has reported that patients’ pain experience is actively related to extensive factors, such as mood changes and affective states [20-23]. Although all our sensory-discriminative measures from GeoPain grouped together, there was a separate assembly composed of VAS pain measurement and mood in our results. Over the course of the study, we noticed a bidirectional association between PANAS positive mood scores and VAS (*P*=.009), but not with PAINS (*P*=.14). Meanwhile, the higher the VAS pain levels reported, the worse the PANAS positive mood scores, and the more positive the patient felt, the lower was his, her, or their VAS. As humans are vastly susceptible to diurnal and seasonal mood variations with work, sleep, and daylength across multiple cultures [24], these mood swings create a large potential for disparities in VAS scores reported by even the same patient along the trial. These disparities became obvious in our study when we analyzed autocorrelations among neighboring time points of VAS scores that showed consistent lower test-retest long-term reliability compared with GeoPain’s PAINS. Hence, varying degrees of altered mood in patients could lead to inaccuracy in the VAS-based results from one time point to another in clinical pain trials. It is not a surprise that many medications that improve or stabilize mood, such as antidepressants (eg, tricyclic antidepressant), are widely considered effective for the treatment of chronic pain conditions; and perhaps their success in clinical pain trials is *in part* explained by their additional indirect effect on VAS scoring. On the other hand, the minimal association of GeoPain’s measurements with mood suggests that they have better sensitivity for evaluating treatment effect over time for sensory-discriminative pain.

### Worsening in Pain Area Is Linked to Chronification, Not Visual Analog Scale Score

In addition to pain intensity, a crucial component of pain processing in the peripheral and central nervous system is area extension of the pain. In our study, the spread of pain from its epicenter in our TMD patient group was significantly correlated with the years of their pain suffering, not their overall intensity on the VAS score. This is frequently seen in pain or more specifically in migraine neuroimaging studies that show neuroplasticity at the functional, structural, and molecular levels. Hence, the impact of pain chronification is linked to its area extension, which might be associated with the progression of central sensitization. For instance, molecular studies with positron emission tomography in chronic patients in vivo have shown that there is a dysfunction in the endogenous mu-opioid system that is highly related to years of pain or more specifically in migraine suffering and PAINS score [14-16]. This is arguably one of the main analgesic systems in our brains.

To address the clinical and research conundrum above, multiple groups have developed questionnaires with 2D body map tools and required patients to delineate the pain area, as a cross, checkmark, or score by counting large body regions affected. Attempts to analyze such recorded data are still too subjective and serve the purpose of general assumptions of patients’ clinical pain complexity. Some studies have addressed the complexity of pain evaluation in the clinical setting [25-28]. For instance, one study indicated that the complexity of chronic pain in a biopsychosocial context includes not only physical but also mental and social outcomes [25]. Another study explored the accuracy of the questionnaire painDETECT to detect neuropathic components of orofacial pain when compared with a reference standard of clinical diagnosis. According to the results of that study, painDETECT, as well as other generic screening tools, must be adapted and revalidated specifically for orofacial pain patients [26]. Our results reinforce the need for more detailed and intuitive scoring of 3D pain area and intensity combined, even within body subregions such as GeoPain’s PAINS, which provides a better assessment of the sensory-discriminative pain severity status quo in real time.

### Show Me Where Your Pain Area and Intensity Number Summation Are, and I Will Tell You Where Your Treatment Is Working

In the era of precision medicine, pain treatments have become more focused and personalized. Consequently, more accurate maps to assess pain and its response to specific therapies are needed. Our clinical trial was a well-fitted example; we used high-definition neuromodulation of the patients’ unilateral motor cortex, purposely in the craniofacial homuncular region. The results demonstrated that compared with VAS, PAINS provided a much higher power of analysis with 2 or 3 times less number of patients needed, depending on the craniofacial subregion analyzed⸺both sides, ipsilateral or contralateral side to the cortical neuromodulation. As reported previously [13], the sensitivity of the GeoPain score was able to detect differences between active and sham groups in the first week of treatment, instead of only after 1 month with VAS.

The motor and sensory homuncular representations from our bodies are extremely accurate, especially from the trigeminal nervous system [12]. The cells and coordinates of GeoPain’s grid system are based on multiple neuroanatomical landmarks, facilitating the translation back from the app to the patient’s own body of the subregion affected by dictating the pain location in medical terms, by therapeutically targeting the region (eg, trigger point injection), by challenging it with quantitative sensory testing, with precise homuncular matching at individual and group levels [11]. Further pixilation of the grid is possible, but it loses the neurophysiological and clinical meaning. On the other hand, tracking pain changes via the traditional single VAS scoring in the overall body or even for large regions would not be sensitive enough to detect those changes within the subregions and in reasonable time. Figuratively, it would be as if driving using a map with only the country or state borders depicted, but without any local road descriptions or coordinates. This deficient GPS is even more inefficient when managing chronic pain patients suffering from multiple pain disorders, such as concurrent migraine and fibromyalgia, undergoing different pain therapies or not (eg, monoclonal antibodies that target calcitonin gene-related peptide and pregabalin). The extension of the patient’s pain comorbidities could cloud the evaluation of the severity of each pain disorder (eg, head and full body pain) and the precise analgesic effect and subregional site of action from a new pain therapy in a clinical trial.

### Conclusions

GeoPain measures exhibited great precision for capturing severity at baseline and treatment effect over time of sensory-discriminative pain compared with the traditional VAS score, which was highly modulated by mood. Further studies with different pain disorders and treatments are needed to confirm our validation results that were based on our previous clinical translational studies. Nonetheless, GeoPain is a valid, consistent, and reliable 3D mobile app for tracking, analyzing, and communicating pain at the individual and group levels.

## Appendix

Multimedia Appendix 1 Flowchart of the patients included in the clinical trial.

Multimedia Appendix 2 Demographic data of the patients included in the clinical trial.

## Abbreviations

HD-tDCS: high-definition transcranial direct current stimulation
M1: putative primary cortex
NRS: numerical rating scale
PAINS: pain area and intensity number summation
PANAS: positive and negative affect schedule
PC1: first principal component
PC2: second principal component
PCA: principal component analysis
TMD: temporomandibular disorder
VAS: visual analog scale
