# Supplementary figures and images for: Sensory-Discriminative Three-Dimensional Body Pain Mobile App Measures Versus Traditional Pain Measurement With a Visual Analog Scale: Validation Study
Source: JMIR Mhealth Uhealth. 2020 Aug 19;8(8):e17754. doi: 10.2196/17754 (PMC7468641; doi:10.2196/17754)

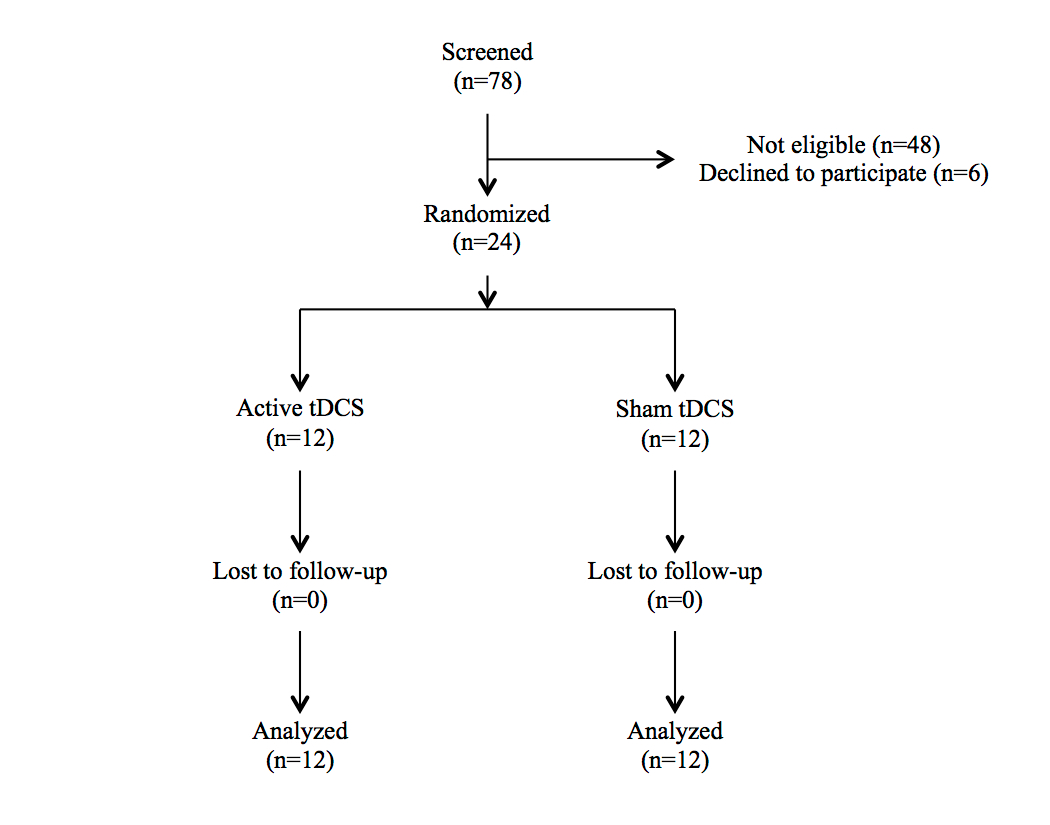

Supplement: Multimedia Appendix 1 [file mhealth_v8i8e17754_app1.png]

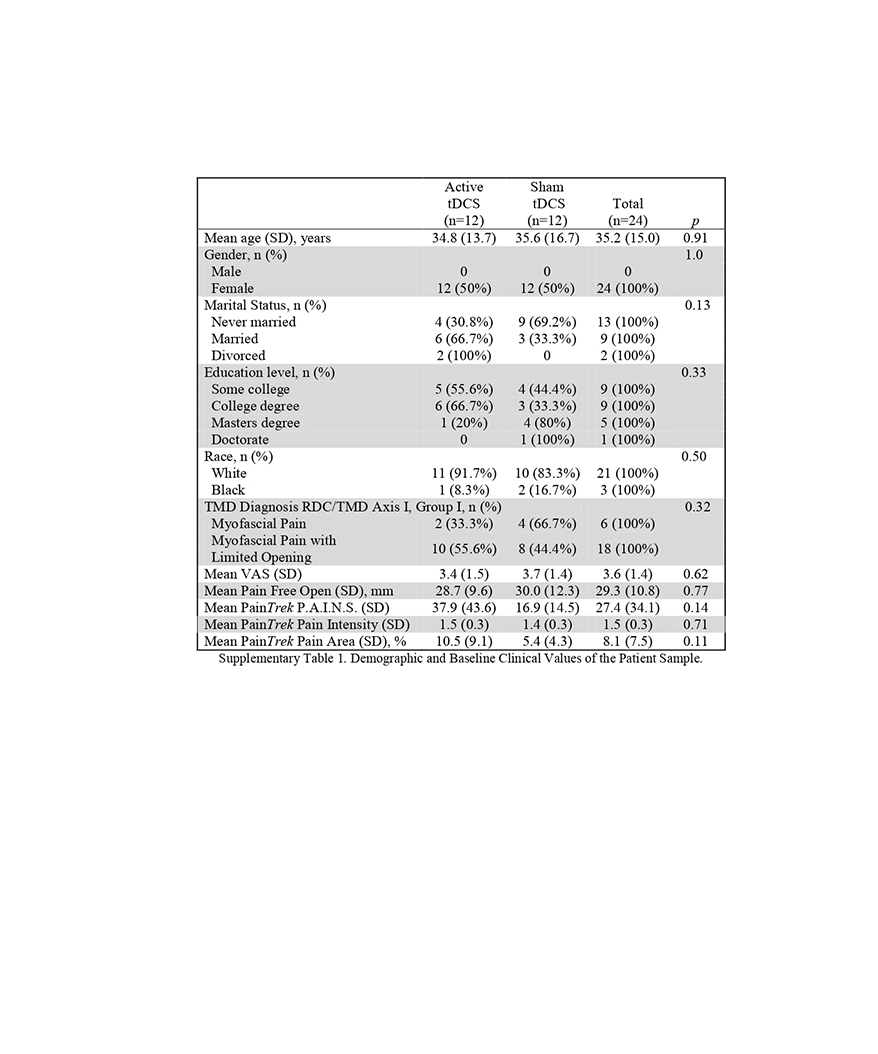

Supplement: Multimedia Appendix 2 [file mhealth_v8i8e17754_app2.png]
